# Outcomes and Discriminatory Accuracy of the CHA_2_DS_2_VASc Score in Atrial Fibrillation and Cancer

**DOI:** 10.1016/j.jacadv.2023.100609

**Published:** 2023-09-16

**Authors:** Waqas Ullah, Mathew DiMeglio, Daniel R. Frisch, Rodrigo Bagur, Louise Y. Sun, David L. Fischman, Andrija Matetic, Bonnie Ky, Mamas A. Mamas

**Affiliations:** a Department of Cardiology, Thomas Jefferson University Hospitals, Philadelphia, Pennsylvania, USA; b Department of Cardiology, London Health Sciences Centre, Western University, London, Ontario, Canada; c Division of Cardiothoracic Anesthesiology, Department of Anesthesiology, Perioperative and Pain Medicine, Stanford University School of Medicine, Palo Alto, California, USA; d Department of Cardiology, University Hospital of Split, Split, Croatia; e Department of Cardiology, University of Pennsylvania, Philadelphia, Pennsylvania, USA; f Department of Cardiology, Keele Cardiovascular Research Group, Keele University, Keele, United Kingdom

**Keywords:** anticoagulation, atrial fibrillation, bleeding, stroke

## Abstract

**Background:**

Atrial fibrillation (AF) is highly prevalent among cancer patients. The role of traditional risk stratification scores in the context of different cancer types in these patients remains unknown.

**Objectives:**

The purpose of this study was to determine the discriminative accuracy of the CHA_2_DS_2_VASc score for ischemic stroke using receiver operating characteristic and area under the curve.

**Methods:**

The National Readmission Database (2015-2019) was used to identify all AF patients stratified by the cancer diagnosis, type, and CHA_2_DS_2_VASc category (low; moderate; high risk). Outcomes at 30-day readmission were compared between cancer and noncancer groups using hierarchical multivariable logistic regression to calculate adjusted odds ratios (aORs) and 95% CIs.

**Results:**

A total of 6,996,088 AF patients were identified at index admission. Of these, 4,242,630 (642,237 cancer, 3,600,393 noncancer) were readmitted at 30 days. Cancer patients (92.1%) had a higher proportion of high CHA_2_DS_2_VASc scores compared with their noncancer counterparts (89.8%, *P* < 0.001). The 30-day readmission rate and incidence of major bleeding in cancer patients were significantly higher compared with their corresponding noncancer group across all CHA_2_DS_2_VASc categories. Among the different cancer types, hematological and lung cancer had a high propensity for major bleeding. The odds of ischemic stroke were lower in the cancer group across high (1.9% vs 2.4%; aOR: 0.78; 95% CI: 0.76-0.79; *P* < 0.0001), moderate (0.8% vs 1.3%; aOR: 0.57; 95% CI: 0.50-0.64; *P* < 0.0001), and low (0.4% vs 0.9%; aOR: 0.46; 95% CI: 0.34-0.62; *P* < 0.0001) risk category relative to the noncancer group irrespective of type of cancer. CHA_2_DS_2_VASc category had a statistically significant discriminatory accuracy for ischemic stroke in both cancer and noncancer patients.

**Conclusions:**

Cancer patients with AF are at a higher risk of readmission and major bleeding. The risk of ischemic stroke during readmission appears to be lower than noncancer patients. These findings may have implications for anticoagulant therapy in cancer patients.

The prevalence of atrial fibrillation (AF) among patients with cancer ranges from 2.5% to 4% at the time of cancer diagnosis and follow-up, and up to about 30% in surgically treated cancer patients.^1^ A recent meta-analysis observed that the overall prevalence of AF in cancer patients can be up to 47% compared with their noncancer counterparts.^2^ The higher AF prevalence observed in cancer may reflect neoplastic infiltration of cardiac tissues, mechanical and metabolic effects on the heart, and adverse effects of neoplastic chemotherapies, radiotherapy and thoracic surgeries.^3^^,^^4^

AF in the general population is associated with a nearly 5-fold higher age-adjusted independent risk of ischemic stroke.^5^ This risk is further compounded by the hypercoagulable state of cancer. Current guidelines recommend the use of the well-validated CHA_2_DS_2_VASc score as a discrimination tool for risk stratification of ischemic stroke in the general population with AF to guide the provision of anticoagulant therapy.^6^ However, the utility of this score in patients with active cancer has not been validated. Moreover, while some studies have linked new-onset AF in cancer patients with poor in-hospital outcomes, there has been no study specifically addressing the risk of major outcomes such as stroke, major bleeding, or all-cause readmission of cancer patients at 30 days.^7^ To bridge this knowledge gap, the current study aimed to identify the impact of AF on the risk of developing ischemic stroke and major bleeding at 30 days among the most common cancer types stratified by CHA_2_DS_2_VASc score. Furthermore, the predictive ability of different CHA_2_DS_2_VASc categories for stroke risk discrimination among cancer types was also explored.

## Methods

### Data source

Data were obtained from the National Readmission Database (NRD). NRD is a publicly available all-payer database of the United States that is closely monitored by the Healthcare Cost and Utilization Project (HCUP) and was established by the Agency for Healthcare Research and Quality. NRD includes information on >7 million hospitalizations/y, representing more than 35 million weighted discharges annually from 28 states. NRD comprises discharge and readmission records of 58.2% of all U.S. hospitalizations. Data from NRD are anonymized and hence exempted from the approval of Institutional Review Boards or ethics committees (https://www.hcup-us.ahrq.gov/nrdoverview.jsp).

### Selection criteria

The nationally weighted 2015 to 2019 NRD claims were utilized to select the 30-day readmission data of all U.S. adult patients (>18 years) with an admitting diagnosis of AF. The standard International Classification of Disease, Clinical Modifications codes were used to identify the population of interest (Supplemental Table 1). Using the special variable “NRD visit-link,” index admission of the individual cases was identified. The variables “HOSP-NRD,” “NRD_Striatum,” and “DISCWTS” were used for clustering, stratification, and weighting of data, respectively. The “NRD days_to_event” and “length_of_stay” variables were used to calculate the readmission day of the same population. As NRD is annualized and only patients admitted within the same calendar year could be identified, we sequentially included the first 11-month data from each year to ensure all patients have a 30-day follow-up. In compliance with HCUP regulations, observations with a cell count <11 were not reported.

### Comparison groups

The study sample was stratified based on the risk of ischemic stroke determined by the CHA_2_DS_2_VASc. The CHA_2_DS_2_VASc score was categorized into 3 groups: low risk (CHA_2_DS_2_VASc = 0 in males and CHA_2_DS_2_VASc = 1 in females), moderate risk (CHA_2_DS_2_VASc = 1 in males and CHA_2_DS_2_VASc = 2 in females), and high risk (CHA_2_DS_2_VASc >2 in males and CHA_2_DS_2_VASc >3 in females). Each CHA_2_DS_2_VASc category was compared under 2 major groups: patients with any type of cancer and those without a diagnosis of cancer. The former group was divided into colorectal cancer, lung cancer, breast cancer, prostate cancer, hematological cancer, and ‘other’ cancer types.

### Study outcomes

The major outcomes were to compare the association between CHA_2_DS_2_VASc category and 30-day all-cause readmission rate, ischemic stroke, and major bleeding among cancer vs noncancer patients.

### Statistical analysis

Categorical data were reported in percentages for each comparison group and were compared using the Pearson chi-square test. Continuous data were presented as mean ± SD and median (IQR). After assessing for distribution of data, continuous variables were compared using the independent *t*-test analysis (for normally distributed) or the Mann-Whitney U test for nonnormally distributed data. A binomial multivariable logistic regression model (enter algorithm) was created to estimate adjusted odds ratios (aORs) for 30-day ischemic stroke, major bleeding, and readmission rate. An adjustment was performed for the following variables: hospital bed size, hospital location/teaching status, hospital region, weekend admission, primary expected payer, smoking, dyslipidemia, anemia, thrombocytopenia, chronic renal failure, liver disease, and coagulopathy. Sensitivity analysis by selecting patients who were on long-term anticoagulant therapy was also performed to assess its impact on ischemic stroke and major bleeding. The performance of CHA_2_DS_2_VASc risk score and CHA_2_DS_2_VASc categories in predicting the risk of ischemic stroke during readmission across different cancer types was determined using receiver operating characteristic. An area under the curve (AUC) for both cancer and noncancer groups was calculated to assess how well the logistic regression model fits the data set. A *P* value <0.05 was regarded as statistically significant. SPSS 27 software (IBM Corp) and R version 4.3 were used for all statistical analysis.

## Results

### Selection of cases

From September 2015 to November 2019, a total of 6,996,088 weighted samples of AF patients were identified at index admission. Of these, 5,991,736 (85%) had no active cancer, while 1,004,351 (15%) had a diagnosis of any active cancer. The most common cancer type was hematologic (n = 156,044, 15.5%) followed by lung (n = 122,524, 12.2%), prostate (n = 58,392, 5.8%), breast (n = 31,777, 3.2%), and colorectal (n = 11,557, 1.2%). Other cancer types contributed 62.1% of the total patient population (Supplemental Figure 1). The proportion of major cancer types stratified by CHA_2_DS_2_VASc category at 30-day readmission is presented in Supplemental Table 2.

### Baseline characteristics

**Figure 1:**
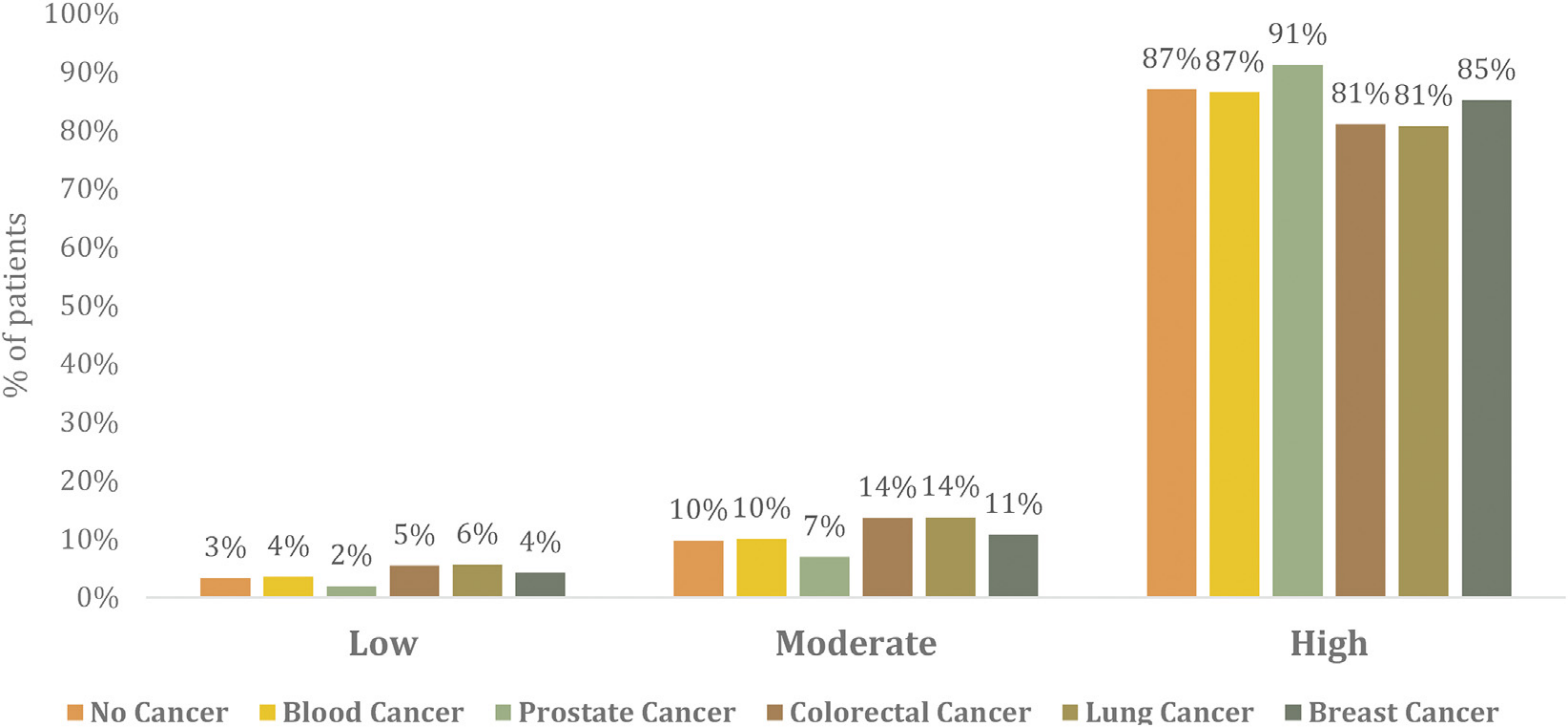
**Proportion of Patients With Each CHA**_**2**_**DS**_**2**_**VASc Score Category by Cancer Type**

**Figure 2:**
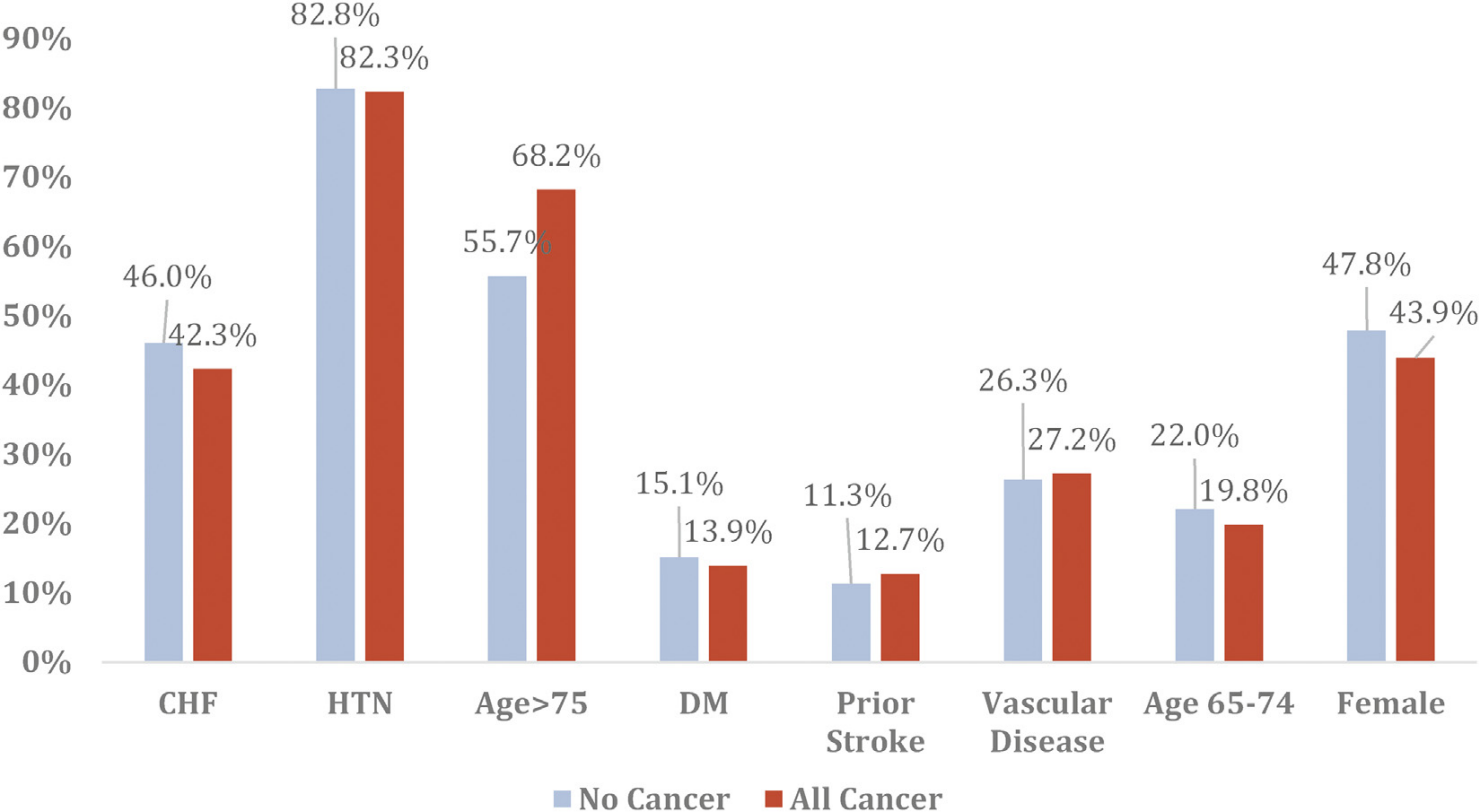
**Evaluation of the CHA**_**2**_**DS**_**2**_**VASc Risk Score Components Based on Cancer Status** CHF = congestive heart failure; DM = diabetes mellitus; HTN = hypertension.

The included population was stratified by CHA_2_DS_2_VASc category. The noncancer group was compared with overall cancer patients and with one of the aforementioned cancer subtypes at the level of each CHA_2_DS_2_VASc category. Among the overall cancer group, the most common CHA_2_DS_2_VASc designation was high risk (n = 916,038, 91.2%) followed by moderate risk (n = 68,891, 6.9%) and low risk (n = 19,422, 1.9%). A similar distribution was observed in the noncancer cohort with a large proportion designated as high risk (n = 5,210,197, 87.0%) followed by moderate (n = 581,831, 9.7%) and low risk (n = 199,708, 3.3%). Prostate cancer exhibited the highest proportion of high-risk patients (91%), while lung and colorectal cancer exhibited the highest proportion of low-risk patients (6% and 5%, respectively) (Figure 1). The most common contributors to CHA_2_DS_2_VASc score were history of hypertension (82.3% vs 82.8%), age >75 years (68.2% vs 55.7%), congestive heart failure (42.3% vs 46.0%), and female sex (43.9% vs 47.8%) in the cancer and noncancer cohort, respectively (Figure 2). The detailed distribution of demographics, hospital characteristics, primary payer information, risk of mortality, and baseline characteristics at 30-day readmission are given in Supplemental Table 3.

### Analysis of major outcomes across CHA_2_DS_2_VASc risk category at 30-day readmission

Cancer patients experienced significantly higher adjusted odds of all-cause 30-day readmission in the high (64.6% vs 62.0%; aOR: 1.04; 95% CI: 1.03-1.04; *P* < 0.0001), moderate (56.6% vs 48.1%; OR: 1.17; 95% CI: 1.16-1.18; *P* < 0.0001), and low (58.7% vs 42.5%; aOR: 1.37; 95% CI: 1.36-1.39; *P* < 0.0001) risk category compared with noncancer patients. Despite the higher rates of readmission, the odds of 30-day readmission with ischemic stroke events was lower in the cancer group throughout the high (1.9% vs 2.4%; aOR: 0.78; 95% CI: 0.76-0.79; *P* < 0.0001), moderate (0.8% vs 1.3%; aOR: 0.57; 95% CI: 0.50-0.64; *P* < 0.0001), and low (0.4% vs 0.9%; aOR: 0.46; 95% CI: 0.34-0.62; *P* < 0.0001) risk category relative to the noncancer group (Supplemental Figure 5, Table 1). In contrast, the adjusted odds of major bleeding were significantly higher in the cancer cohort across the high (2.2% vs 2.0%; aOR: 1.08; 95% CI: 1.06-1.10; *P* < 0.0001), moderate (2.8% vs 1.9%; aOR: 1.40; 95% CI: 1.31-1.49; *P* < 0.0001), and low (2.8% vs 1.9%; aOR: 1.33; 95% CI: 1.17-1.50; *P* < 0.0001) risk categories compared with the corresponding noncancer cohort.

**Table 1:** Comparison of In-Hospital Ischemic Stroke and Major Bleeding According to the CHA_2_DS_2_VASc Score Categories at 30 Days

	Low Risk				Moderate Risk				High Risk			
	No CancerCount	CancerCount	OR (95% CI)	*P* Value	No CancerCount	CancerCount	OR (95% CI)	*P* Value	No CancerCount	CancerCount	OR (95% CI)	*P* Value
Readmission	85,069 (42.5%)	11,410 (58.7%)	1.37 (1.36-1.39)	<0.0001	280,307 (48.1%)	39,046 (56.6%)	1.17 (1.16-1.18)	<0.0001	3,235,017 (62.0%)	591,781 (64.6%)	1.04 (1.03-1.04)	<0.0001
Stroke	750 (0.90%)	48 (0.40%)	0.46 (0.34-0.62)	<0.0001	3,664 (1.30%)	310 (0.80%)	0.57 (0.50-0.64)	<0.0001	77,151 (2.40%)	11,456 (1.90%)	0.78 (0.76-0.79)	<0.0001
Major bleeding	1,656 (1.90%)	315 (2.80%)	1.33 (1.17-1.50)	<0.0001	5,379 (1.90%)	1,110 (2.80%)	1.40 (1.31-1.49)	<0.0001	65,492 (2.00%)	13,259 (2.20%)	1.08 (1.06-1.10)	<0.0001

Values are n (%) unless otherwise indicated. Low-risk group indicates CHA_2_DS_2_VASc=0 in males and CHA_2_DS_2_VASc = 1 in females; low-moderate group indicates CHA_2_DS_2_VASc = 1 in males and CHA_2_DS_2_VASc = 2 in females; moderate-high group indicates CHA_2_DS_2_VASc ≥2 in males and CHA_2_DS_2_VASc ≥3 in females.

CHA_2_DS_2_VASc risk score–risk score composed of the following components: congestive heart failure, arterial hypertension, age cutoffs (65-75 and ≥75 years), diabetes mellitus, previous stroke, vascular disease and sex category.

### Outcomes at 30-day readmission stratified by risk and cancer types

#### Ischemic stroke events

Among those designated as low risk by CHA_2_DS_2_VASc score, there were similar adjusted odds of stroke across all cancer types relative to the noncancer cohort. In the moderate risk category, the adjusted odds of stroke were significantly lower in hematological (0.6% vs 1.3%; aOR: 0.66; 95% CI: 0.50-0.87; *P* = 0.004), prostate (0.3% vs 1.3%; aOR: 0.34; 95% CI: 0.15-0.76; *P* = 0.009), and breast cancer (0.5% vs 1.2%; aOR: 0.45; 95% CI: 0.25-0.82; *P* = 0.009) compared with patients without a diagnosis of cancer, respectively. The high risk CHA_2_DS_2_VASc category showed that all cancer types had a significantly lower risk of ischemic stroke at 30 days compared with noncancer patients after their first admitting diagnosis of AF. The proportion and adjusted odds of stroke in hematological (1.5% vs 2.3%; aOR: 0.73; 95% CI: 0.69-0.77; *P* < 0.0001), prostate (1.8% vs 2.3%; aOR: 0.90; 95% CI: 0.83-0.93; *P* = 0.02), breast (1.9% vs 2.3%; aOR: 0.81; 95% CI: 0.73-0.90; *P* < 0.0001), lung (1.6% vs 2.3%; aOR: 0.66; 95% CI: 0.62-0.70; *P* < 0.0001), and colorectal cancer (1.5% vs 2.3%; aOR: 0.66; 95% CI: 0.54-0.80; *P* < 0.0001) relative to their noncancer counterparts all crossed the threshold of statistical significance (Supplemental Figure 2, Table 2, Central Illustration).

**Table 2:** Outcomes at 30-Day Readmission Stratified by Risk and Cancer Types (Total Patients 4,242,630)

	Major Bleeding				Stroke			
	Cancer	No Cancer	OR (95% CI)	*P* Value	Cancer	No Cancer	OR (95% CI)	*P* Value
Low risk (n = 96,479)								
Blood cancer	158 (3.80%)	1,813 (2.00%)	1.70 (1.35-2.14)	<0.0001	18 (0.40%)	780 (0.80%)	1.00 (0.55-1.81)	0.99
Prostate cancer	<11	1,964 (2.00%)	0.77 (0.36-1.62)	0.49	<11	796 (0.80%)	1.03 (0.26-4.12)	0.97
Breast cancer	16 (2.10%)	1,956 (2.00%)	1.14 (0.69-1.90)	0.60	<11	793 (0.80%)	0.76 (0.31-1.85)	0.55
Lung cancer	295 (6.80%)	1,676 (1.80%)	4.30 (3.76-4.91)	<0.0001	32 (0.70%)	766 (0.80%)	0.98 (0.68-1.40)	0.90
Colorectal cancer	<11	1,970 (2.10%)	0.18 (0.03-0.92)	0.04	<11	798 (0.80%)	1.08 (0.78-1.94)	0.67
Moderate risk (n = 319,353)								
Blood cancer	475 (4.40%)	6,015 (1.90%)	1.80 (1.59-2.04)	<0.0001	63 (0.60%)	3,910 (1.30%)	0.66 (0.50-0.87)	0.004
Prostate cancer	32 (1.60%)	6,458 (2.00%)	0.66 (0.46-1.01)	0.05	<11	3,968 (1.30%)	0.34 (0.15-0.76)	0.009
Breast cancer	41 (2.10%)	6,449 (2.00%)	1.07 (0.78-1.46)	0.67	11 (0.50%)	3,963 (1.20%)	0.45 (0.25-0.82)	0.009
Lung cancer	547 (5.20%)	5,943 (1.90%)	3.05 (2.78-3.34)	<0.0001	129 (1.20%)	3,845 (1.20%)	1.01 (0.85-1.21)	0.89
Colorectal cancer	21 (1.90%)	6,469 (2.00%)	0.89 (0.57-1.38)	0.61	12 (1.10%)	3,962 (1.20%)	1.09 (0.61-1.93)	0.76
High risk (n = 3,826,798)								
Blood cancer	2,863 (2.90%)	75,888 (2.00%)	1.31 (1.26-1.37)	<0.0001	1,442 (1.50%)	87,164 (2.30%)	0.73 (0.69-0.77)	<0.0001
Prostate cancer	762 (2.30%)	77,989 (2.10%)	1.03 (0.96-1.11)	0.33	620 (1.80%)	87,987 (2.30%)	0.90 (0.83-0.98)	0.02
Breast cancer	380 (2.20%)	78,371 (2.10%)	1.08 (0.98-1.20)	0.11	332 (1.90%)	88,275 (2.30%)	0.81 (0.73-0.90)	<0.0001
Lung cancer	3,552 (5.40%)	75,199 (2.00%)	2.83 (2.74-2.94)	<0.0001	1,022 (1.60%)	87,585 (2.30%)	0.66 (0.62-0.70)	<0.0001
Colorectal cancer	98 (1.40%)	78,653 (2.10%)	0.64 (0.53-0.80)	<0.0001	106 (1.50%)	88,501 (2.30%)	0.66 (0.54-0.80)	<0.0001

Values are n (%) unless otherwise indicated.

**Central Illustration:**
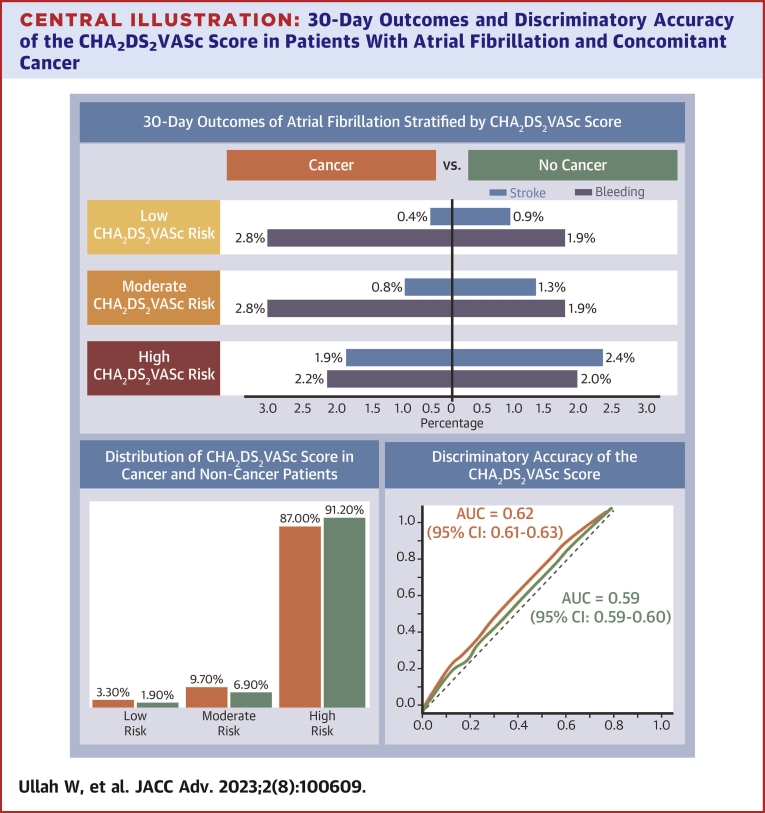
**30-Day Outcomes and Discriminatory Accuracy of the CHA**_**2**_**DS**_**2**_**VASc Score in Patients With Atrial Fibrillation and Concomitant Cancer**

#### Major bleeding events

Among the low-risk CHA_2_DS_2_VASc category, the adjusted odds of major bleeding events were significantly higher in patients with hematological malignancies (3.8% vs 2.0%; aOR: 1.70; 95% CI: 1.35-2.14; *P* < 0.0001) and lung cancer (6.8% vs 1.8%; aOR: 4.30; 95% CI: 3.76-4.91; *P* < 0.0001) compared with the low-risk noncancer group, respectively. Similarly, in moderate-risk patients, blood (4.4% vs 1.9%; aOR: 1.80; 95% CI: 1.59-2.04; *P* < 0.0001) and lung cancer (5.2% vs 1.9%; aOR: 3.05; 95% CI: 2.78-3.34; *P* < 0.0001) was associated with a higher risk of major bleeding compared with patients who had no active cancer, respectively. In the high-risk cohort, the risk of major bleeding remained significantly higher in patients with hematological (2.9% vs 2.0%; aOR: 1.31; 95% CI: 1.26-1.37; *P* < 0.0001) and lung cancer (5.2% vs 1.9%; aOR: 3.05; 95% CI: 2.78-3.34; *P* < 0.0001) compared with the high-risk noncancer cohort, respectively. Patients with prostate and breast cancer continued to have a similar risk of major bleeding compared with the noncancer patients across all CHA_2_DS_2_VASc categories (Supplemental Figure 3).

#### Sensitivity analysis by the long-term use of anticoagulants

The frequency of anticoagulant therapy among different cancer groups stratified by the CHA_2_DS_2_VASc category is presented in Supplemental Figure 4, and Supplemental Table 4. Overall, 37.5% of cancer and noncancer patients were on long-term anticoagulation therapy. With an increase in the CHA_2_DS_2_VASc risk score from low and moderate to high risk, there was an increase in the long-term use of anticoagulants (18.5% and 26.6%-38.6%) in the cancer cohort, and 18.0% vs 26.1% vs 36.3% in the noncancer cohort, respectively. Interestingly, there were significant differences in the prescription of long-term anticoagulants among the cancer types. For instance, only 12.7% and 16.6% of the colorectal cancer patients received anticoagulant therapy compared with 22.0% and 25.6% of the prostate cancer patients among the low and moderate risk CHA_2_DS_2_VASc categories, respectively. Among the high-risk patients, the proportion of anticoagulant therapy use was comparable among all cancer types.

In contrast to the net results, long-term anticoagulant therapy in high-risk patients with breast cancer (aOR: 1.29; 95% CI: 1.12-1.50; *P* = 0.001) had a higher risk of major bleeding compared with a similar cohort of noncancer patients. Similarly, with anticoagulant therapy in moderate- and high-risk categories, there was no significant difference in the risk of stroke among noncancer vs breast and prostate cancer patients. The adjusted odds of major bleeding among patients with hematological and lung cancer on long-term anticoagulant therapy remain higher compared with their noncancer counterparts across all CHA_2_DS_2_VASc categories (Supplemental Table 5).

#### Receiver operating characteristics of CHA_2_DS_2_VASc score and risk category for ischemic stroke across cancer types

Among noncancer patients, statistically significant discriminatory accuracy for ischemic stroke was observed with the CHA_2_DS_2_VASc score (AUC: 0.599; 95% CI: 0.596-0.602; *P* < 0.0001). A similar pattern was observed in the overall cancer cohort with the CHA_2_DS_2_VASc score (AUC: 0.624; 95% CI: 0.617-0.631; *P* < 0.0001) (Supplemental Figure 6). However, significant variation was observed among the different cancer types. The CHA_2_DS_2_VASc category as a predictability model failed to distinguish between the risk for 30-day ischemic stroke among patients with prostate (AUC: 0.527; 95% CI: 0.498-0.555; *P* = 0.079) and colorectal cancer (AUC: 0.558; 95% CI: 0.495-0.621; *P* = 0.1) (Supplemental Figure 7, Supplemental Table 6).

## Discussion

This study evaluated a nationally representative cohort derived from the largest U.S. administrative claims database. The important findings include: 1) Both cancer and noncancer inpatients with a principal admission diagnosis of AF are at a high risk of ischemic stroke as indicated by ∼90% of these patients belonging to a high-risk CHA_2_DS_2_VASc category; 2) the most common contributor to CHA_2_DS_2_VASc score was hypertension in both groups, followed by advanced age (age >75 years) mostly in the cancer group; 3) overall, cancer was associated with a relatively lower 30-day risk of ischemic stroke compared with the noncancer cohort across all CHA_2_DS_2_VASc risk categories; 4) overall, the CHA_2_DS_2_VASc score was predictive for ischemic events in noncancer and all-cancer patients, except for patients with prostate and colorectal cancer; 5) and cancer was associated with a higher 30-day major bleeding events in patients at moderate or high CHA_2_DS_2_VASc risk categories although the overall high bleeding rate in cancer patients was driven by hematological and lung cancer, as there remained no difference in the risk of major bleeding between other cancer types and noncancer cohorts irrespective of the use of anticoagulant therapy and CHA_2_DS_2_VASc category.

Malignancy causes profound alterations in hemostasis leading to an increased propensity toward both thrombotic (ischemic stroke) and bleeding complications.^8^^,^^9^ The former is caused by cancer-related hypercoagulability, noninfectious endocarditis, paradoxical embolization of cancer-related clots, tumor occlusion, and shared risk factors of cancer and ischemic stroke.^10^^,^^11^ Mechanisms driving coagulopathy include cancer-related factors such as cytokine secretion (tumor necrosis factor-α), tissue factor expression, liver metastases, and tumor cell characteristics (mucin production in adenocarcinoma), as well as treatment-related factors such as thrombocytopenia mainly through chemotherapy administration.^9^^,^^12^ While cancer can cause increased rates of both bleeding and thrombosis, previous literature has found that bleeding might be the predominant phenotype in this patient population.^8^ Thus, the balance between the risk of major bleeding and the prevention of cancer-related thrombotic events is of paramount importance in conditions that require antithrombotic therapies such as AF.

Surprisingly, despite a lower utilization of anticoagulation therapy, our study consistently demonstrated a relatively lower 30-day risk of stroke among all subtypes of high-risk cancer patients compared with noncancer patients. This could plausibly be explained by the competing risk of cancer-related mortality in cancer patients, as the NRD does not capture postdischarge mortality in the community settings, we could not account for this in our analyses. A prior study by Jang et al^13^ on the long-term cancer-stroke relationship demonstrated a higher incidence of stroke in cancer patients; however, this risk was not adjusted for the CHA_2_DS_2_VASc category, where patients are more likely to be in the higher risk group. In contrast, other studies have not shown any increased stroke risk associated with cancer although stroke risk associated with individual cancers was not assessed.^14^ Finally, a report from the Nationwide Inpatient Sample reported a lower in-hospital stroke risk in cancer patients admitted with a principal diagnosis of AF.^15^ Subsequent expert opinions rightly identified that most of the prothrombotic factors also increase bleeding risk in cancer patients and that ischemic risks in cancer might be comparable to those of noncancer patients.^11^ Therefore, a dynamic and individualized approach to anticoagulation therapy is required to guide the management of AF in cancer patients.

Prior studies on the predictors of major bleeding in patients with active cancer have shown that older age, female sex, hypertension, and history of prior stroke were directly associated with a higher risk of major bleeding.^16^ The risk was further accentuated with anticoagulant therapy. Interestingly, these predictors are the key elements of the CHA_2_DS_2_VASc score which is designed for the risk stratification of ischemic stroke. Our results support these findings by demonstrating a higher incidence of 30-day major bleeding in cancer compared with noncancer patients, especially in the high-risk CHA_2_DS_2_VASc category. Furthermore, we have identified that among all different cancer types, patients with hematologic and lung cancer have a higher tendency for major bleeding irrespective of the CHA_2_DS_2_VASc category and use of anticoagulation medications. The former could be explained by the disruption of the coagulation cascade and increased fibrinolysis, while in lung cancer increased invasion, metastasis and impaired angiogenesis can be linked to higher bleeding events.^17^ Together, these findings suggest that the type of cancer and CHA_2_DS_2_VASc category should be taken into consideration while treating AF with anticoagulation therapy in these patients.

Our analysis suggests that the CHA_2_DS_2_VASc score was predictive for ischemic events in cancer patients although its performance was modest for most cancers and was nonpredictive for patients with prostate and colorectal cancer. Previous models for the estimation of thromboembolic risk have been notoriously unreliable among cancer patients.^18^^,^^19^ It is also important to note that the development of both the CHADS_2_ and CHA_2_DS_2_VASc score system specifically excluded oncologic patients from analysis.^20^^,^^21^ While many patients with cancer have risk factors included in the CHA_2_DS_2_VASc score, it is suggested that cancer poses an additional thrombotic risk that is not captured in traditional risk prediction models. Studies have shown an increased risk of both thromboembolism and mortality with each point elevation in CHA_2_DS_2_VASc score beyond that of their noncancer counterparts.^19^^,^^21^ However, our results show both CHA_2_DS_2_VASc score and risk category were comparable in accuracy for estimating thromboembolic risk in overall cancer as well as noncancer cohorts. This suggests that risk factors as outlined by traditional prediction tools are the predominant driver of thrombotic risk in this patient population. However, the unequal distribution of thromboembolism among different cancer types highlights the importance of using additional criteria for risk stratification. More importantly, our findings also hinted toward the high CHA_2_DS_2_VASc category being indicative of a higher bleeding risk in cancer patients with AF.

The findings from this study have several important clinical implications. While oncologic patients are often at high risk of thromboembolism, 30-day readmission in this population was predominantly driven by major bleeding events. This highlights the importance of considering additional criteria such as baseline hemoglobin, platelets, coagulation studies, as well as bleeding risk prediction models (ie, HAS-BLED) to better characterize the risk of major bleeding with anticoagulation. Also, the distribution of major bleeding and its relationship to anticoagulant use was found to be highly dependent on the type of malignancy with breast cancer posing the highest risk with anticoagulation while hematological and lung cancer featured an augmented risk of bleeding irrespective of anticoagulation. Lastly, our study affirms the utility of CHA_2_DS_2_VASc as a thromboembolic risk prediction tool among cancer patients, but there is significant variation in discrimination according to cancer type with colorectal and prostate cancer performing worse.

### Study Limitations

The findings of our study are constrained by the inherent limitations of the NRD. The duration of AF, choice and reason for an anticoagulation agent, type of chemotherapies, and stages of cancer could not be determined due to the lack of granular data. Similarly, due to insufficient information on the components of bleeding scores, the HAS-BLED risk score could not be calculated. CHA_2_DS_2_VASc score had poor discriminatory accuracy for predicting ischemic stroke in patients with cancers. Although all codes were validated by the recommended HCUP database, the possibility of inadvertent misclassification could not be eliminated. Furthermore, we could not capture data on laboratory parameters (coagulation parameters, hematological indices, kidney, and liver function tests) and disease severity that could have played a role in the pooled estimates. Although a logistic regression model was used to account for potential effect modifiers, the impact of unknown and unmeasurable covariates could not be assessed. Given the annualized nature of data, we excluded patients who were admitted in December of each included year. Due to insufficient long-term follow-up data, we could only report the 30-day outcomes of the index admission with AF and cancer.

## Conclusions

Cancer patients with concomitant AF are at a higher risk of major bleeding events and readmission at 30 days of the index admission with AF. This risk was driven by patients with hematologic and lung cancer and was independent of the CHA_2_DS_2_VASc score and the use of anticoagulant therapy. Decisions about anticoagulation should be considered in light of these findings. Cancer patients appeared to have a lower 30-day stroke probably due to a survivorship bias and competing risk of cancer-related mortality. The performance of the CHA_2_DS_2_VASc risk category was significantly different between different cancer types, with overall worse discrimination of ischemic stroke risk in prostate and colorectal cancer patients. The CHA_2_DS_2_VASc risk score requires further validation in different cancer patients with AF.

PERSPECTIVES **COMPETENCY IN MEDICAL KNOWLEDGE:** Compared with noncancer patients, AF in cancer patients, especially those with lung and hematologic cancers, are at an increased risk of major bleeding and 30-day readmission independent of the CHA_2_DS_2_VASc score and the use of anticoagulation.

**TRANSLATIONAL OUTLOOK:** Future large-scale research with more comprehensive clinical variables and predictors of major bleeding is needed to elucidate further the role of the safety of anticoagulation in AF with cancer.

## Funding support and author disclosures

The authors have reported that they have no relationships relevant to the contents of this paper to disclose.
