# Supplementary material for: Outcomes and Discriminatory Accuracy of the CHA2DS2VASc Score in Atrial Fibrillation and Cancer
Source: JACC Adv. 2023 Sep 16;2(8):100609. doi: 10.1016/j.jacadv.2023.100609 (PMC11198258; doi:10.1016/j.jacadv.2023.100609)

**Supplemental Table 1. ICD 10 codes used for identification of cases, baseline characteristics and outcomes**

| Diagnoses and outcomes | Codes |
| --- | --- |
| Atrial fibrillation | I48.91, I48.20-21, I48.11, I48.19, I48.0 |
| Atrial flutter | I48.3, I48.4, I48.92 |
| Long-term (current) use of anticoagulants | Z79.01 |
| Use of antiplatelets | Z79.02 |
| Dyslipidemia | E78.x |
| Smoker | Z72.0 |
| Cardiac arrest | I46.2 (due to cardiac condition); I46.8 and I46.9 (due to non-cardiac condition) |
| Ischemic cardiomyopathy | I25.5 |
| Thrombocytopenia | D69.4.x, D69.5.x, D69.6.x |
| Chronic renal failure | N18.x |
| Anemias | D62.x, D63.x, D64.x |
| Coagulopathies | D65.x, D66.x, D67.x, D68.x, D69.x |
| Liver disease | K70.x, K72.1.x, K72.9.x, K73.x, K74.x, K75.x, K76.x, K77.x |
| Acute ischemic stroke | I63.x |
| Pericardial effusion | I31.3 |
| Stroke | I630x-I639 |
| Major Bleeding | R58x, L76x, K661, I62x, R04x, |
| CHA2DS2VASc score components | Codes |
| Congestive heart failure | I11.0, I13.0, I13.2, I09.81, I09.9, I25.5, I50.x, I42.x, I43.x |
| Arterial hypertension | I10.x, I11.x, I12.x, I13.x, I14.x, I15.x, I67.4 |
| Diabetes Mellitus | E08.x, E09.x, E10.x, E11.x, E13.x |
| Previous Stroke | Z86.73 |
| Vascular disease | I20.x-I25.x, I70.x, I73.x, Z95.1, Z95.5, Z98.61 |
| Cancer Codes |  |
| Colorectal Cancer | C18, C19, C20, C218 |
| Prostate Cancer | C61 |
| Breast Cancer | C500-C50922 |
| Lung Cancer | C340-C3492 |
| Blood Cancer | C810-C96x |

**Supplemental Table 2: Total number of cancer patients readmitted at 30-days and its risk stratification (percentages are within the risk category)**

|  | Total 4242630 | Low Risk 96479 | Moderate Risk 319353 | High Risk 3826798 |
| --- | --- | --- | --- | --- |
| No Cancer | 3600393 | 85069 (88.2%) | 280307 (87.8%) | 3235017 (84.5%) |
| All Cancer | 642237 | 11410 (11.8%) | 39046 (12.2%) | 591781 (15.5%) |
| Blood Cancer | 112675 | 4127 (4.3%) | 10878 (3.4%) | 97671 (2.6%) |
| Prostate Cancer | 36219 | 472 (0.5%) | 1971 (0.6%) | 33775 (0.9%) |
| Breast Cancer | 19818 | 746 (0.8%) | 1981 (0.6%) | 17092 (0.4%) |
| Lung Cancer | 80645 | 4368 (4.5%) | 10485 (3.3%) | 65792 (1.7%) |
| Colorectal Cancer | 8839 | 440 (0.5%) | 1112 (0.3%) | 7287 (0.2%) |
| Other Cancers | 384041 | 1257 (1.3%) | 12619 (4.0%) | 370164 (9.7%) |

**Supplemental Table 3: Baseline characteristics of patients readmitted at 30-days with atrial fibrillation stratified by cancer types**

|  | No Cancer | All Cancer | Blood Cancer | Prostate Cancer | Breast Cancer | Lung Cancer | Colorectal Cancer |
| --- | --- | --- | --- | --- | --- | --- | --- |
|  | 3600393 | 642237 | 112675 | 36219 | 19818 | 80645 | 8839 |
| Sex | | | | | | | |
| Male | 1860014 (51.7%) | 361475 (56.3%) | 69366 (61.6%) | 36219 (100.0%) | 425 (2.1%) | 47781 (59.2%) | 5738 (64.9%) |
| Female | 1740379 (48.3%) | 280762 (43.7%) | 43309 (38.4%) | <11 | 19393 (97.9%) | 32864 (40.8%) | 3100 (35.1%) |
| Admission Day | | | | | | | |
| Weekday | 2793249 (77.6%) | 502117 (78.2%) | 88812 (78.8%) | 28344 (78.3%) | 15704 (79.2%) | 64395 (79.9%) | 7139 (80.8%) |
| Weekend | 807144 (22.4%) | 140119 (21.8%) | 23863 (21.2%) | 7875 (21.7%) | 4114 (20.8%) | 16250 (20.1%) | 1700 (19.2%) |
| Admission Type | | | | | | | |
| Elective | 3211101 (89.3%) | 567748 (88.6%) | 99531 (88.5%) | 31947 (88.4%) | 17561 (88.8%) | 67948 (84.4%) | 7123 (80.7%) |
| Emergent | 382865 (10.7%) | 73313 (11.4%) | 12922 (11.5%) | 4201 (11.6%) | 2209 (11.2%) | 12560 (15.6%) | 1699 (19.3%) |
| Bed Size | | | | | | | |
| Small | 649387 (18.0%) | 109561 (17.1%) | 18121 (16.1%) | 6449 (17.8%) | 3782 (19.1%) | 13226 (16.4%) | 1411 (16.0%) |
| Medium | 1019596 (28.3%) | 179099 (27.9%) | 29008 (25.7%) | 10200 (28.2%) | 5749 (29.0%) | 21950 (27.2%) | 2431 (27.5%) |
| Large | 1931410 (53.6%) | 353576 (55.1%) | 65546 (58.2%) | 19569 (54.0%) | 10287 (51.9%) | 45468 (56.4%) | 4996 (56.5%) |
| Patient Location | | | | | | | |
| Central | 811976 (22.6%) | 142389 (22.2%) | 25159 (22.4%) | 9425 (26.1%) | 5269 (26.6%) | 16791 (20.9%) | 2079 (23.6%) |
| Fringe | 961275 (26.8%) | 193506 (30.2%) | 33279 (29.6%) | 10031 (27.7%) | 5606 (28.3%) | 22592 (28.1%) | 2354 (26.7%) |
| Metro >250K | 795823 (22.2%) | 135834 (21.2%) | 23965 (21.3%) | 7364 (20.4%) | 4000 (20.2%) | 17751 (22.0%) | 1796 (20.3%) |
| Metro >50 | 374613 (10.4%) | 65745 (10.2%) | 11519 (10.2%) | 3517 (9.7%) | 1898 (9.6%) | 8540 (10.6%) | 893 (10.1%) |
| Micro | 355590 (9.9%) | 57752 (9.0%) | 10038 (8.9%) | 3136 (8.7%) | 1704 (8.6%) | 7819 (9.7%) | 945 (10.7%) |
| None | 292327 (8.1%) | 46219 (7.2%) | 8570 (7.6%) | 2690 (7.4%) | 1322 (6.7%) | 7026 (8.7%) | 758 (8.6%) |
| Teaching Status | | | | | | | |
| MNT | 855098 (23.8%) | 145579 (22.7%) | 22814 (20.2%) | 7753 (21.4%) | 4187 (21.1%) | 17995 (22.3%) | 1913 (21.6%) |
| MT | 2381454 (66.1%) | 441935 (68.8%) | 81438 (72.3%) | 25289 (69.8%) | 13982 (70.6%) | 56014 (69.5%) | 6129 (69.3%) |
| NMH | 363841 (10.1%) | 54722 (8.5%) | 8423 (7.5%) | 3177 (8.8%) | 1649 (8.3%) | 6636 (8.2%) | 797 (9.0%) |
| Hospital Designation | | | | | | | |
| LMA | 1890343 (52.5%) | 360070 (56.1%) | 64481 (57.2%) | 20761 (57.3%) | 11494 (58.0%) | 42509 (52.7%) | 4796 (54.3%) |
| SMA | 1346209 (37.4%) | 227444 (35.4%) | 39770 (35.3%) | 12281 (33.9%) | 6675 (33.7%) | 31500 (39.1%) | 3246 (36.7%) |
| MIA | 270170 (7.5%) | 42643 (6.6%) | 6468 (5.7%) | 2345 (6.5%) | 1305 (6.6%) | 5360 (6.6%) | 635 (7.2%) |
| NUR | 93671 (2.6%) | 12080 (1.9%) | 1956 (1.7%) | 833 (2.3%) | 345 (1.7%) | 1276 (1.6%) | 161 (1.8%) |
| Primary Payer | | | | | | | |
| None | 2972041 (82.6%) | 568719 (88.6%) | 94537 (84.0%) | 32157 (88.9%) | 16877 (85.2%) | 65034 (80.7%) | 6949 (78.6%) |
| Medicare | 190225 (5.3%) | 13143 (2.0%) | 2956 (2.6%) | 616 (1.7%) | 711 (3.6%) | 4121 (5.1%) | 474 (5.4%) |
| Medicaid | 337203 (9.4%) | 49092 (7.6%) | 13008 (11.6%) | 2715 (7.5%) | 2009 (10.1%) | 9249 (11.5%) | 1216 (13.8%) |
| Private Insurance | 35278 (1.0%) | 2187 (0.3%) | 503 (0.4%) | 91 (0.3%) | 70 (0.4%) | 569 (0.7%) | 61 (0.7%) |
| Self-Pay | 4978 (0.1%) | 251 (0.0%) | 59 (0.1%) | 12 (0.0%) | <11 | 53 (0.1%) | <11 |
| No Charge | 57545 (1.6%) | 8432 (1.3%) | 1535 (1.4%) | 599 (1.7%) | 124 (0.6%) | 1576 (2.0%) | 132 (1.5%) |
| Household Income | | | | | | | |
| 0-25th | 1016601 (28.6%) | 149756 (23.6%) | 24423 (21.9%) | 8420 (23.5%) | 5025 (25.7%) | 22324 (28.0%) | 2279 (26.1%) |
| 26th-50th | 993958 (28.0%) | 171194 (27.0%) | 29875 (26.8%) | 9245 (25.8%) | 5284 (27.0%) | 22281 (27.9%) | 2395 (27.4%) |
| 51st-75th | 877223 (24.7%) | 165448 (26.1%) | 29783 (26.7%) | 9292 (26.0%) | 5027 (25.7%) | 19823 (24.9%) | 2339 (26.8%) |
| 76th-100th | 668298 (18.8%) | 148536 (23.4%) | 27261 (24.5%) | 8844 (24.7%) | 4236 (21.6%) | 15327 (19.2%) | 1716 (19.7%) |
| Risk of Mortality | | | | | | | |
| No Class Specified | 301 (0.0%) | 53 (0.0%) | <11 | <11 | <11 | 11 (0.0%) | <11 |
| Minor | 317305 (8.8%) | 38710 (6.0%) | 1754 (1.6%) | 663 (1.8%) | 266 (1.3%) | 2258 (2.8%) | 563 (6.4%) |
| Moderate | 1259743 (35.0%) | 227517 (35.4%) | 37726 (33.5%) | 9699 (26.8%) | 6248 (31.5%) | 14414 (17.9%) | 2898 (32.8%) |
| Major | 1474614 (41.0%) | 287030 (44.7%) | 53416 (47.4%) | 20232 (55.9%) | 10390 (52.4%) | 49491 (61.4%) | 4016 (45.4%) |
| Extreme | 548429 (15.2%) | 88927 (13.8%) | 19774 (17.5%) | 5622 (15.5%) | 2912 (14.7%) | 14472 (17.9%) | 1361 (15.4%) |
| Severity of Illness | | | | | | | |
| No Class Specified | 301 (0.0%) | 53 (0.0%) | <11 | <11 | <11 | 11 (0.0%) | <11 |
| Minor | 225589 (6.3%) | 32444 (5.1%) | 1428 (1.3%) | 1223 (3.4%) | 925 (4.7%) | 1690 (2.1%) | 331 (3.7%) |
| Moderate | 1193948 (33.2%) | 210478 (32.8%) | 25582 (22.7%) | 10824 (29.9%) | 6040 (30.5%) | 20434 (25.3%) | 2815 (31.8%) |
| Major | 1615413 (44.9%) | 304267 (47.4%) | 60315 (53.5%) | 18922 (52.2%) | 9732 (49.1%) | 41375 (51.3%) | 4258 (48.2%) |
| Extreme | 565143 (15.7%) | 94995 (14.8%) | 25345 (22.5%) | 5247 (14.5%) | 3120 (15.7%) | 17135 (21.2%) | 1434 (16.2%) |
| Comorbidities | | | | | | | |
| Hypertension | 3050271 (84.7%) | 535109 (83.3%) | 87094 (77.3%) | 29437 (81.3%) | 16079 (81.1%) | 60670 (75.2%) | 6940 (78.5%) |
| Diabetes Mellitus | 552384 (15.3%) | 90968 (14.2%) | 14111 (12.5%) | 4998 (13.8%) | 3177 (16.0%) | 11664 (14.5%) | 1319 (14.9%) |
| Acute Kidney Injury | 968355 (26.9%) | 166905 (26.0%) | 34046 (30.2%) | 11170 (30.8%) | 4590 (23.2%) | 14455 (17.9%) | 2328 (26.3%) |
| Cardiac Arrest | 20301 (0.6%) | 2339 (0.4%) | 457 (0.4%) | 155 (0.4%) | 67 (0.3%) | 301 (0.4%) | 31 (0.4%) |
| Pericardial Effusion | 47322 (1.3%) | 9634 (1.5%) | 2491 (2.2%) | 406 (1.1%) | 553 (2.8%) | 3494 (4.3%) | 100 (1.1%) |
| Cardiac Tamponade | 7070 (0.2%) | 1463 (0.2%) | 399 (0.4%) | 54 (0.1%) | 125 (0.6%) | 910 (1.1%) | <11 |
| Alcohol Use | 14174 (0.4%) | 1027 (0.2%) | 105 (0.1%) | 73 (0.2%) | 15 (0.1%) | 144 (0.2%) | 19 (0.2%) |
| Blood Loss Anemia | 53149 (1.5%) | 11194 (1.7%) | 1560 (1.4%) | 762 (2.1%) | 245 (1.2%) | 863 (1.1%) | 470 (5.3%) |
| CHF | 1873768 (52.0%) | 295642 (46.0%) | 49067 (43.5%) | 15279 (42.2%) | 8520 (43.0%) | 25748 (31.9%) | 2966 (33.6%) |
| Coagulopathy | 332450 (9.2%) | 68287 (10.6%) | 20567 (18.3%) | 4256 (11.7%) | 1792 (9.0%) | 6747 (8.4%) | 799 (9.0%) |
| COPD | 1293342 (35.9%) | 222122 (34.6%) | 31619 (28.1%) | 9865 (27.2%) | 6048 (30.5%) | 51131 (63.4%) | 2301 (26.0%) |
| Depression | 491805 (13.7%) | 81168 (12.6%) | 13327 (11.8%) | 3181 (8.8%) | 3120 (15.7%) | 9748 (12.1%) | 862 (9.8%) |
| Drug USe | 97879 (2.7%) | 8504 (1.3%) | 1525 (1.4%) | 494 (1.4%) | 259 (1.3%) | 1639 (2.0%) | 158 (1.8%) |
| Electrolyte Abnormalities | 1447828 (40.2%) | 254639 (39.6%) | 50622 (44.9%) | 15159 (41.9%) | 8803 (44.4%) | 33756 (41.9%) | 4025 (45.5%) |
| HIV | 4638 (0.1%) | 703 (0.1%) | 288 (0.3%) | 27 (0.1%) | 7 (0.0%) | 114 (0.1%) | 24 (0.3%) |
| Hypothyroidism | 728014 (20.2%) | 140425 (21.9%) | 22778 (20.2%) | 4648 (12.8%) | 4776 (24.1%) | 12556 (15.6%) | 1227 (13.9%) |
| Liver Disease | 182749 (5.1%) | 24265 (3.8%) | 4753 (4.2%) | 1225 (3.4%) | 735 (3.7%) | 2704 (3.4%) | 395 (4.5%) |
| Neurologic Disorder | 238662 (6.6%) | 34657 (5.4%) | 5129 (4.6%) | 1908 (5.3%) | 861 (4.3%) | 3525 (4.4%) | 297 (3.4%) |
| Obesity | 723961 (20.1%) | 93178 (14.5%) | 14484 (12.9%) | 4443 (12.3%) | 3951 (19.9%) | 8963 (11.1%) | 1384 (15.7%) |
| Paralysis | 89320 (2.5%) | 12407 (1.9%) | 1835 (1.6%) | 717 (2.0%) | 383 (1.9%) | 1328 (1.6%) | 133 (1.5%) |
| PVD | 434613 (12.1%) | 76762 (12.0%) | 10574 (9.4%) | 4703 (13.0%) | 1680 (8.5%) | 10403 (12.9%) | 838 (9.5%) |
| Psychosis | 46523 (1.3%) | 4293 (0.7%) | 616 (0.5%) | 207 (0.6%) | 182 (0.9%) | 583 (0.7%) | 49 (0.6%) |
| PUD | 504408 (14.0%) | 83653 (13.0%) | 13829 (12.3%) | 3837 (10.6%) | 2781 (14.0%) | 9786 (12.1%) | 911 (10.3%) |
| Renal Failure | 1318363 (36.6%) | 234132 (36.5%) | 41673 (37.0%) | 13463 (37.2%) | 5465 (27.6%) | 16613 (20.6%) | 2290 (25.9%) |
| Rheumatoid Arthritis | 153143 (4.3%) | 25075 (3.9%) | 4465 (4.0%) | 800 (2.2%) | 833 (4.2%) | 2803 (3.5%) | 185 (2.1%) |
| Weight Loss | 292591 (8.1%) | 61506 (9.6%) | 14477 (12.8%) | 4370 (12.1%) | 2216 (11.2%) | 13763 (17.1%) | 1526 (17.3%) |
| All MI | 227754 (6.3%) | 32745 (5.1%) | 5214 (4.6%) | 2138 (5.9%) | 761 (3.8%) | 3035 (3.8%) | 404 (4.6%) |
| PCI | 75066 (2.1%) | 10328 (1.6%) | 1143 (1.0%) | 579 (1.6%) | 162 (0.8%) | 578 (0.7%) | 109 (1.2%) |
| CABG | 59811 (1.7%) | 7345 (1.1%) | 892 (0.8%) | 445 (1.2%) | 67 (0.3%) | 251 (0.3%) | 79 (0.9%) |
| Prior PCI | 43210 (1.2%) | 8066 (1.3%) | 1099 (1.0%) | 431 (1.2%) | 145 (0.7%) | 853 (1.1%) | 66 (0.8%) |
| Prior CABG | 406621 (11.3%) | 73922 (11.5%) | 9755 (8.7%) | 4886 (13.5%) | 923 (4.7%) | 6658 (8.3%) | 651 (7.4%) |
| Prior MI | 403018 (11.2%) | 72218 (11.2%) | 9747 (8.7%) | 3884 (10.7%) | 1373 (6.9%) | 7973 (9.9%) | 841 (9.5%) |
| Vascular Diseases | 814142 (22.6%) | 144764 (22.5%) | 20953 (18.6%) | 9005 (24.9%) | 3114 (15.7%) | 16917 (21.0%) | 1527 (17.8%) |
| Smoking | 1359345 (37.8%) | 278727 (43.4%) | 40436 (35.9%) | 14921 (41.2%) | 6073 (30.6%) | 55772 (69.2%) | 3696 (41.8%) |
| Dyslipidemia | 1918759 (53.3%) | 349802 (54.5%) | 53518 (47.5%) | 19360 (53.5%) | 9466 (47.8%) | 39856 (49.4%) | 4117 (46.6%) |
| CAD | 1594939 (44.3%) | 275306 (42.9%) | 40723 (36.1%) | 16467 (45.5%) | 5640 (28.5%) | 29697 (36.8%) | 2967 (33.6%) |
| Family Hx of CAD | 202699 (5.6%) | 38958 (6.1%) | 5817 (5.2%) | 1714 (4.7%) | 958 (4.8%) | 3955 (4.9%) | 451 (5.1%) |
| Thrombocytopenia | 250999 (7.0%) | 57549 (9.0%) | 21070 (18.7%) | 3375 (9.3%) | 1595 (8.0%) | 6369 (7.9%) | 636 (7.2%) |
| Long Term Anticoagulant | 1263572 (35.1%) | 240667 (37.5%) | 34131 (30.3%) | 12268 (33.9%) | 6852 (34.6%) | 21660 (26.9%) | 2516 (28.5%) |
| Long Term Antiplatelet | 168825 (4.7%) | 30014 (4.7%) | 3760 (3.3%) | 1525 (4.2%) | 641 (3.2%) | 2992 (3.7%) | 302 (3.4%) |

**Note:** Low risk group indicates CHA_2_DS_2_VASc=0 in males and CHA_2_DS_2_VASc=1 in females; low-moderate group indicates CHA_2_DS_2_VASc=1 in males and CHA_2_DS_2_VASc=2 in females; moderate-high group indicates CHA_2_DS_2_VASc≥2 in males and CHA_2_DS_2_VASc≥3 in females.

**Abbreviations:** CABG – coronary artery bypass graft; CAD – coronary artery disease; LMA: Large metropolitan area, SMA: Small metropolitan area MiA: Micropolitan areas, NUR: Non-urban residual, Central: "Central" counties of metro areas of >=1 million population, Fringe: "Fringe" counties of metro areas of >=1 million population, Metro >250K: Counties in metro areas of 250,000-999,999 population, Metro >50: Counties in metro areas of 50,000-249,999 population, Micro: Micropolitan counties, None: Not metropolitan or micropolitan counties, Minor: Minor likelihood of dying, Moderate: Moderate likelihood of dying,Major: Major likelihood of dying: Extreme Extreme likelihood of dying, MNT: Metropolitan Non-Teaching, MT: Metropolitan Teaching, NMH: Non-Metropolitan Hospital, PCD: Pulmonary circulation disorder, CHF: Congestive heart failure, RA: Rheumatoid arthritis, HIV: Human immunodeficiency virus, COPD: Chronic obstructive pulmonary disease; PVD: Peripheral Vascular Disease, PUD: Peptic Ulcer Disease, CHA_2_DS_2_VASc risk score – risk score composed of the following components: CHF, hypertension, age cut-offs (65-75 and ≥75 years), diabetes mellitus, previous stroke, vascular disease and sex category; CVA – cerebrovascular accidents; IHD – ischemic heart disease; IQR – interquartile range; USD – United States Dollar.

**Supplemental Table 4: Frequency of patients on antithrombotic therapies stratified by cancer type and risk of stroke. (percentages are within the respective cancer types)**

|  | No Anticoagulation (2738392) | Anticoagulation (1504238) |
| --- | --- | --- |
| Low Risk | 79034 | 17445 |
| No Cancer | 69736 (82.0%) | 15333 (18.0%) |
| All Cancer | 9298 (81.5%) | 2112 (18.5%) |
| Blood Cancer | 3576 (86.7%) | 551 (13.3%) |
| Prostate Cancer | 369 (78.0%) | 103 (22.0%) |
| Breast Cancer | 606 (81.3%) | 140 (18.7%) |
| Lung Cancer | 3747 (85.8%) | 621 (14.2%) |
| Colorectal Cancer | 384 (87.3%) | 56 (12.7%) |
| Moderate Risk | 235722 | 83631 |
| No Cancer | 207063 (73.9%) | 73244 (26.1%) |
| All Cancer | 28659 (73.4%) | 10387 (26.6%) |
| Blood Cancer | 8647 (79.5%) | 2231 (20.5%) |
| Prostate Cancer | 1467 (74.4%) | 505 (25.6%) |
| Breast Cancer | 1509 (76.2%) | 472 (23.8%) |
| Lung Cancer | 8575 (81.8%) | 1910 (18.2%) |
| Colorectal Cancer | 927 (83.4%) | 185 (16.6%) |
| High Risk | 2423636 | 1403162 |
| No Cancer | 2060023 (63.7%) | 1174994 (36.3%) |
| All Cancer | 363613 (61.4%) | 228168 (38.6%) |
| Blood Cancer | 66321 (67.9%) | 31350 (32.1%) |
| Prostate Cancer | 22116 (65.5%) | 11659 (34.5%) |
| Breast Cancer | 10852 (63.5%) | 6240 (36.5%) |
| Lung Cancer | 46663 (70.9%) | 19129 (29.1%) |
| Colorectal Cancer | 5012 (68.8%) | 2275 (31.2%) |

**Supplemental Table 5: Outcomes at 30-day readmission of patients on long term anticoagulation stratified by risk and cancer types (total patients 1504238)**

|  |  |  |  |  |  |  |  |  |
| --- | --- | --- | --- | --- | --- | --- | --- | --- |
|  | Cancer | No Cancer | Odds Ratio | P-Value | Cancer | No Cancer | Odds Ratio | P-Value |
| Low Risk (17,444) | | | | | | | |  |
| All Cancer | 50 (2.40%) | 360 (2.30%) | 1.00 (0.70-1.42) | 0.99 | <11 | 123 (0.80%) | 0.39 (0.15-1.03) | 0.39 |
| Blood Cancer | 14 (2.50%) | 396 (2.60%) | 1.01 (1.0-1.92) | 0.04 | <11 | 127 (0.80%) | - | - |
| Prostate Cancer | <11 | 410 (2.40%) | - | - | <11 | 127 (0.70%) | - | - |
| Breast Cancer | <11 | 406 (2.30%) | 1.16 (0.40-3.36) | 0.77 | <11 | 127 (0.70%) | - | - |
| Lung Cancer | 47 (7.50%) | 363 (2.20%) | 3.49 (2.51-4.83) | <0.0001 | <11 | 118 (0.70%) | 2.72 (1.36-5.44) | 0.005 |
| Colorectal Cancer | <11 | 410 (2.40%) | - | - | <11 | 127 (0.70%) | - | - |
| Moderate Risk (83,631) | | | | | |  |  | |
| All Cancer | 277 (2.70%) | 1590 (2.20%) | 1.01 (0.86-1.18) | 0.87 | 76 (0.70%) | 836 (1.10%) | 0.72 (0.55-0.93) | 0.01 |
| Blood Cancer | 84 (3.80%) | 1783 (2.20%) | 1.63 (1.25-2.13) | <0.0001 | <11 | 902 (1.10%) | 0.55 (0.27-1.09) | 0.08 |
| Prostate Cancer | 11 (2.20%) | 1856 (2.20%) | 0.90 (0.49-1.67) | 0.76 | <11 | 911 (1.10%) | - | - |
| Breast Cancer | <11 | 1861 (2.20%) | 0.63 (0.28-1.41) | 0.27 | <11 | 910 (1.10%) | 0.32 (0.06-1.49) | 0.15 |
| Lung Cancer | 107 (5.60%) | 1760 (2.20%) | 2.89 (2.35-3.57) | <0.0001 | 38 (2.00%) | 873 (1.10%) | 1.91 (1.37-2.67) | <0.0001 |
| Colorectal Cancer | <11 | 1857 (2.20%) | 2.63 (1.38-5.04) | 0.01 | <11 | 909 (1.10%) | 1.54 (0.44-5.50) | 0.5 |
| High Risk (1,403,163) | | | | | |  |  | |
| All Cancer | 5665 (2.50%) | 26671 (2.30%) | 1.03 (0.99-1.06) | 0.05 | 3738 (1.60%) | 22594 (1.90%) | 0.87 (0.83-0.90) | <0.0001 |
| Blood Cancer | 935 (3.00%) | 31400 (2.30%) | 1.19 (1.10-1.28) | <0.0001 | 417 (1.30%) | 25916 (1.90%) | 0.81 (0.72-0.89) | <0.0001 |
| Prostate Cancer | 287 (2.50%) | 32048 (2.30%) | 0.99 (0.87-1.12) | 0.89 | 211 (1.80%) | 26122 (1.90%) | 1.09 (0.95-1.25) | 0.23 |
| Breast Cancer | 185 (3.00%) | 32151 (2.30%) | 1.29 (1.12-1.50) | 0.001 | 109 (1.80%) | 26223 (1.90%) | 0.92 (0.75-1.11) | 0.36 |
| Lung Cancer | 1190 (6.20%) | 31145 (2.30%) | 2.83 (2.66-3.01) | <0.0001 | 318 (1.70%) | 26015 (1.90%) | 0.89 (0.79-0.99) | 0.04 |
| Colorectal Cancer | 33 (1.50%) | 32303 (2.30%) | 0.59 (0.41-0.84) | 0.59 | 30 (1.30%) | 26303 (1.90%) | 1.04 (1.01-1.06) | 0.001 |

**Supplemental Table 6: Area under the curve (AUC) and its 95% confidence interval to assess the predictability of model for stroke**

| Test Result Variable(s) | AUC | Lower Bound | Upper Bound | Sig (p-value) |
| --- | --- | --- | --- | --- |
| No Cancer | | | | |
| CHADSVASc Score | 0.599 | 0.596 | 0.602 | <0.0001 |
| CHADSVASc Category | 0.524 | 0.521 | 0.526 | <0.0001 |
| All Cancer | | | | |
| CHADSVASc Score | 0.624 | 0.617 | 0.631 | <0.0001 |
| CHADSVASc Category | 0.524 | 0.517 | 0.531 | <0.0001 |
| Blood Cancer | | | | |
| CHADSVASc Score | 0.647 | 0.627 | 0.667 | <0.0001 |
| CHADSVASc Category | 0.537 | 0.518 | 0.555 | <0.0001 |
| Prostate Cancer | | | | |
| CHADSVASc Score | 0.607 | 0.578 | 0.637 | <0.0001 |
| CHADSVASc Category | 0.527 | 0.498 | 0.555 | 0.079 |
| Breast Cancer | | | | |
| CHADSVASc Score | 0.641 | 0.604 | 0.679 | 0.604 |
| CHADSVASc Category | 0.543 | 0.506 | 0.58 | 0.037 |
| Lung Cancer | | | | |
| CHADSVASc Score | 0.607 | 0.584 | 0.631 | <0.0001 |
| CHADSVASc Category | 0.53 | 0.508 | 0.551 | 0.01 |
| Colorectal Cancer | | | | |
| CHADSVASc Score | 0.669 | 0.602 | 0.736 | <0.0001 |
| CHADSVASc Category | 0.558 | 0.495 | 0.621 | 0.1 |

**Supplemental Figure 1: Flow diagram of selection of cases at the index admission.**

**
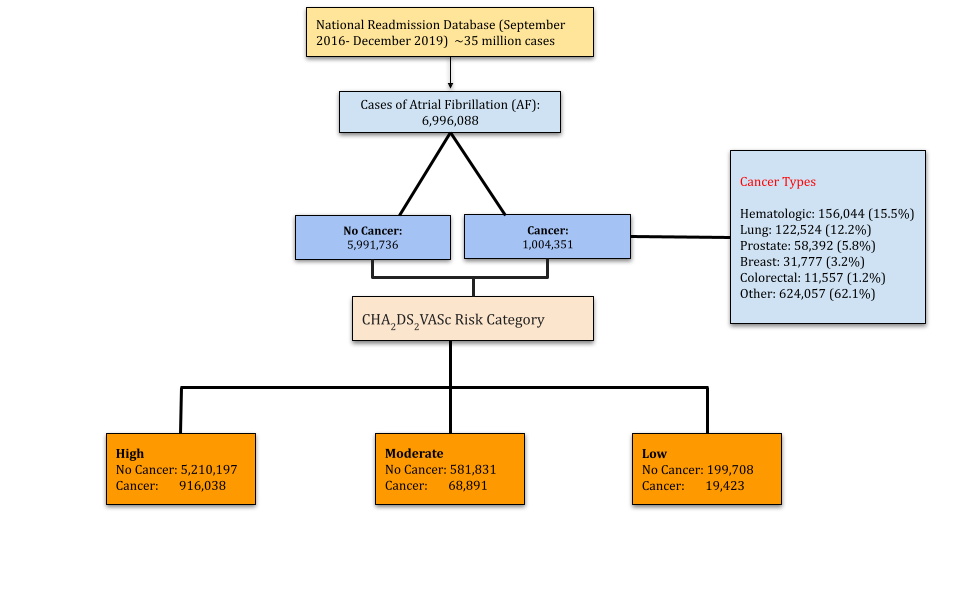
**

**Supplemental Figure 2.** Evaluation of in-hospital ischemic stroke by the CHA_2_DS_2_VASc score categories and cancer types at 30-day readmission.

**
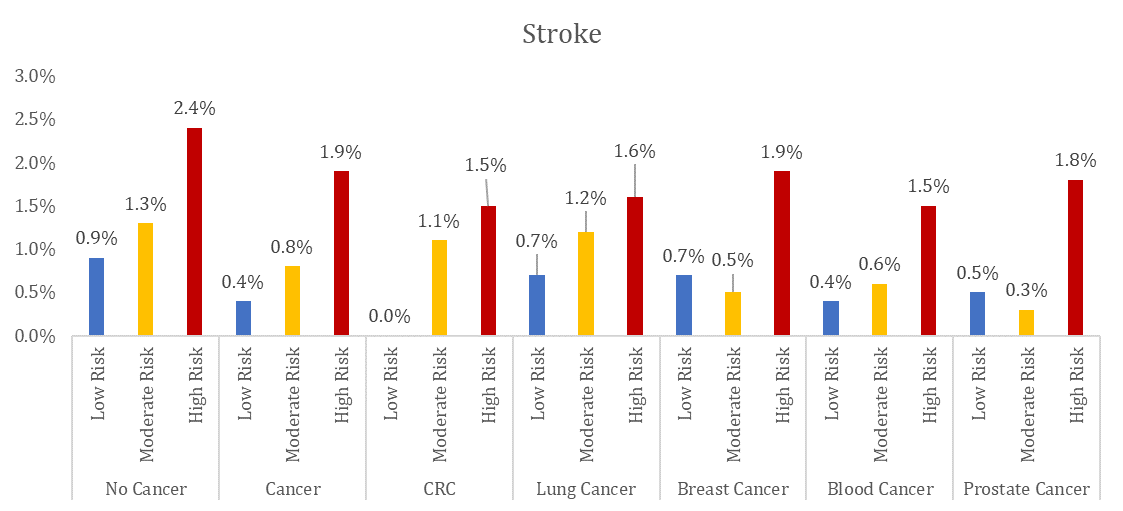
**

**Supplemental Figure 3.** Evaluation of in-hospital major bleeding by the CHA_2_DS_2_VASc score categories and cancer types at 30-day readmission.

**
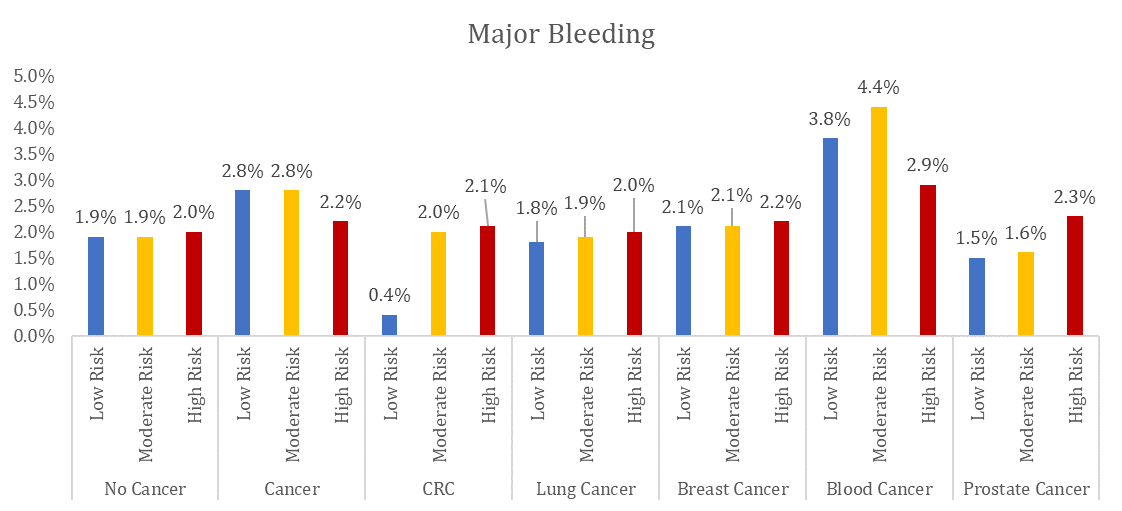
**

**Supplemental Figure 4.** Long-term use of anticoagulants based on the CHA_2_DS_2_VASc score categories and cancer status.

**
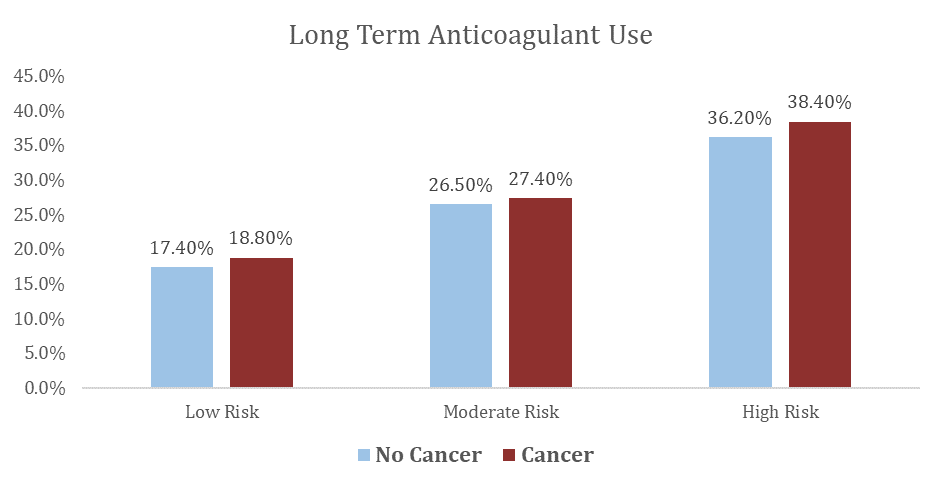
**

**Supplemental Figure 5.** Long-term use of anticoagulants based on the CHA_2_DS_2_VASc score categories and cancer types

**
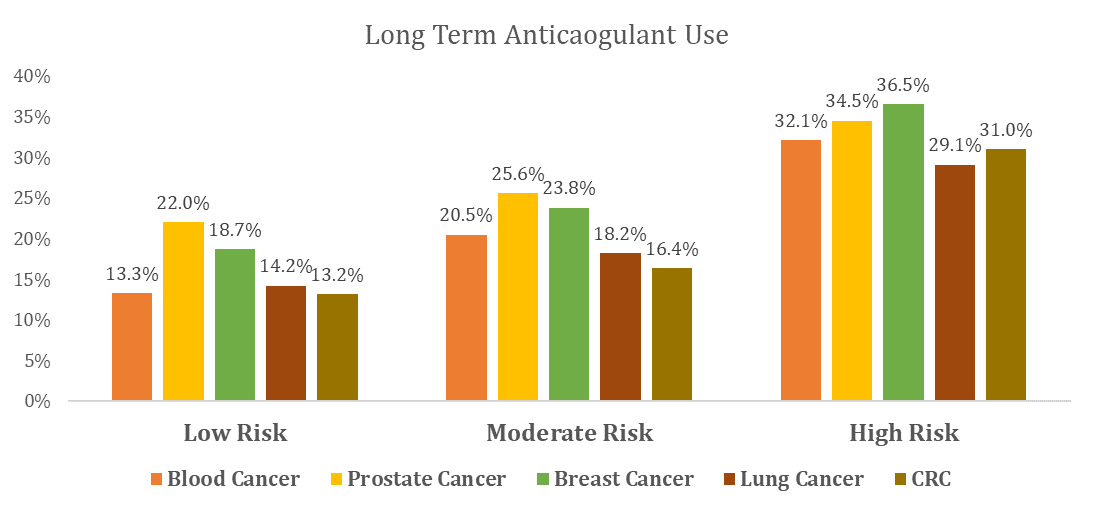
**

**Abbreviations:** CHA_2_DS_2_VASc risk score – risk score composed of the following components: congestive heart failure, arterial hypertension, age cut-offs (65-75 and ≥75 years), diabetes mellitus, previous stroke, vascular disease and sex category; TE – thromboembolism; TIA – transitory ischemic attack.

**Supplemental Figure 6.** Receiver operating characteristics of CHA_2_DS_2_VASc risk score for acute ischemic stroke based on cancer status.

**
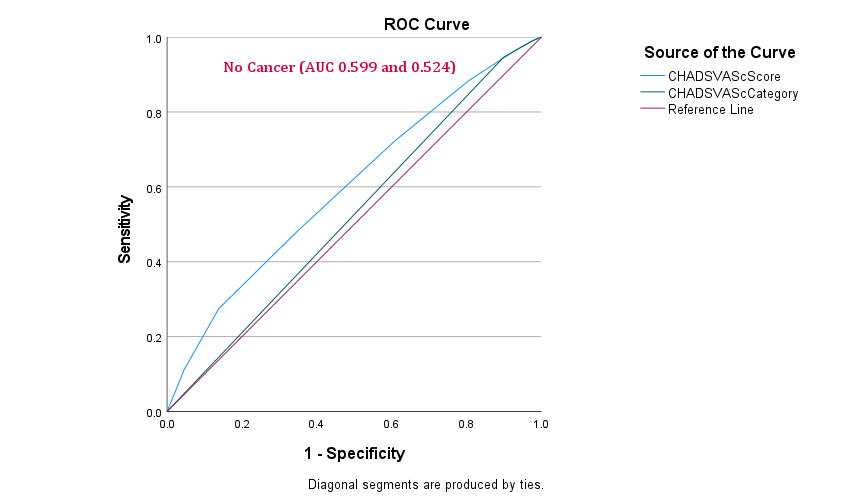

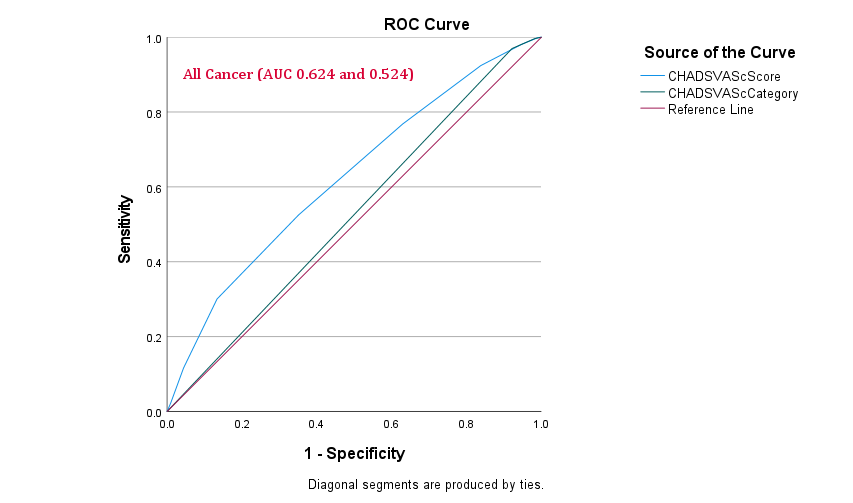
**

**Supplemental Figure 7.** Receiver operating characteristics of CHA_2_DS_2_VASc risk score for stroke based on different cancer types.


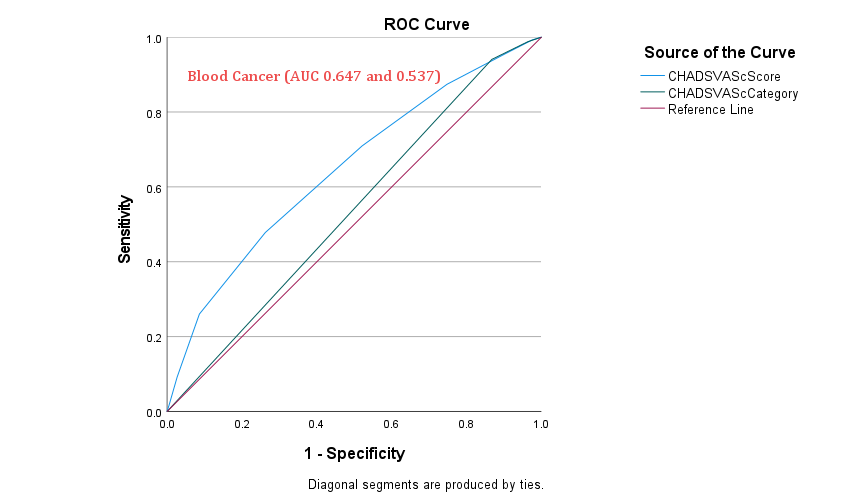

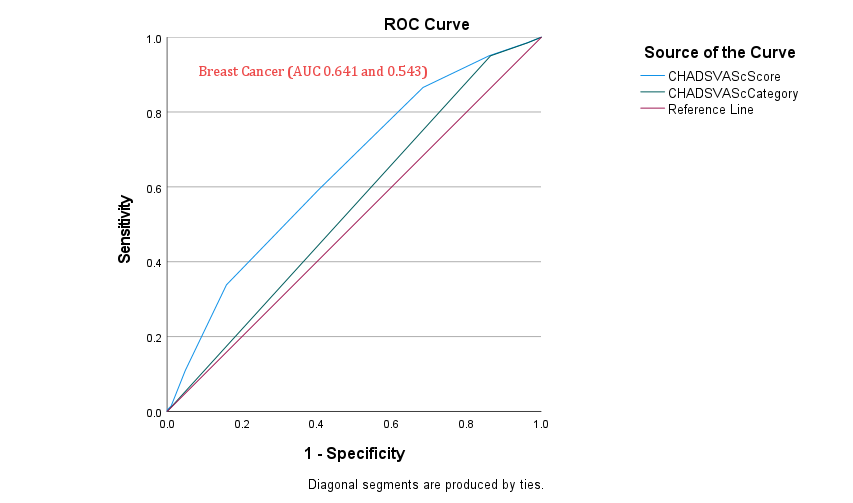


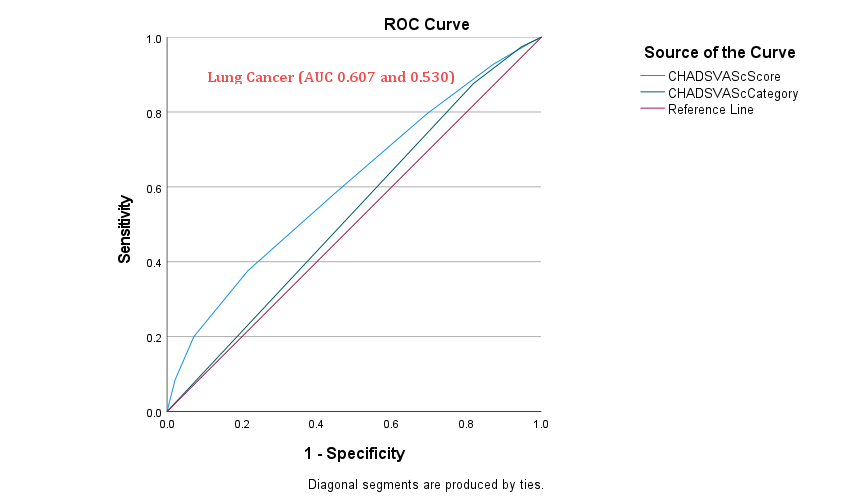

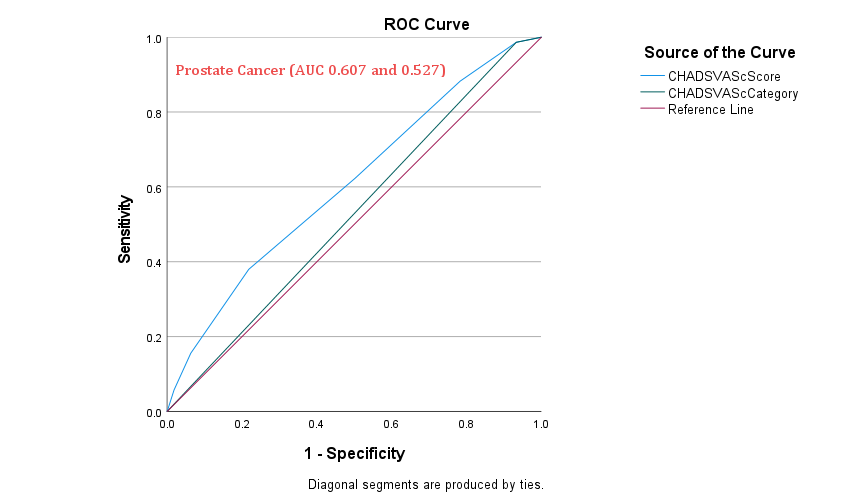


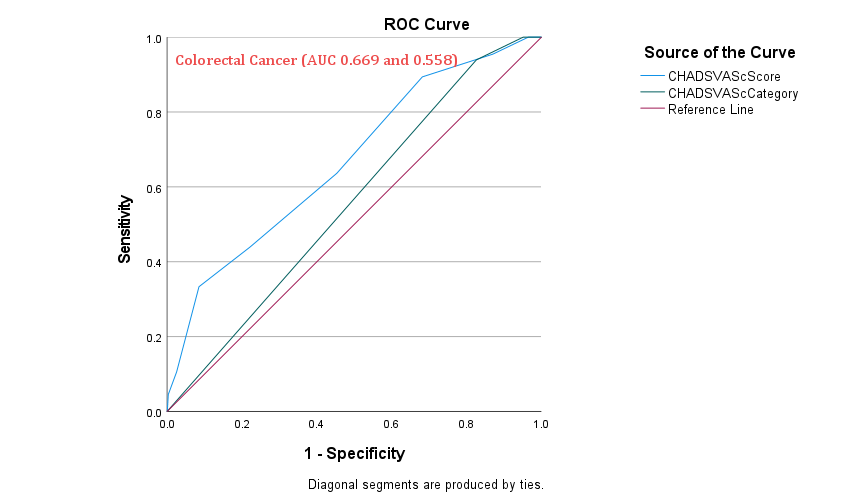

Supplement: Supplementary Tables 1–6, and Supplementary Figures 1–7 [file mmc1.docx]
